# Factors Associated With Health Literacy In Older Korean Adults: Korean National Health And Nutrition Survey 2023

**DOI:** 10.1093/geroni/igaf122.2457

**Published:** 2025-12-31

**Authors:** Eun Ju Park, Jae Jun Lee, Layoung Kim, Gwang Suk Kim

**Affiliations:** Yonsei University College of Nursing, Seoul, Republic of Korea; Yonsei University College of Nursing, Seoul, Republic of Korea; The University of Suwon, Hwaseong, Republic of Korea; Yonsei University College of Nursing, Seoul, Republic of Korea

## Abstract

Health literacy (HL) is essential for managing health and accessing healthcare, yet low HL among older adults is linked to poor health outcomes and disparities. As Korea’s population ages rapidly, understanding the factors influencing HL is crucial for developing effective public health strategies. This study aimed to assess the HL status of older Korean adults and identify the influencing factors using data from the Korean National Health and Nutrition Survey 2023. A total of 1,441 individuals aged 65 and older were included after excluding cases with missing data. HL was measured using self-reported questions developed in Korea, comprising ten items rated on a 4-point Likert scale and one knowledge assessment item. Independent variables included demographic, socioeconomic, and physical and mental health-related factors. Multiple linear regression analysis identified significant predictors of HL. The mean participant age was 72.22±5.00 years, and 55% were female. The average HL score was 27.61±5.63, with only 25.9% correctly answering the knowledge item. Higher education, urban residence, higher income, spousal status, private health insurance, and employment were positively associated with HL, while older age and longer working hours were negatively associated. This study highlights significant disparities in HL among older adults, with sociodemographic and occupational factors playing crucial roles. As the workforce ages, more older adults may engage in long working hours, limiting their access to health information and potentially reducing HL. Given HL’s impact on health behaviors, ensuring appropriate rest for older workers, along with targeted educational interventions, is essential to improving HL.

